# Successful thoracoscopic treatment for tracheoesophageal fistula and esophageal atresia of communicating bronchopulmonary foregut malformation group IB with dextrocardia: a case report of VACTERL association

**DOI:** 10.1186/s40792-020-01099-y

**Published:** 2021-01-06

**Authors:** Toshio Harumatsu, Tatsuru Kaji, Ayaka Nagano, Mayu Matsui, Masakazu Murakami, Koshiro Sugita, Makoto Matsukubo, Satoshi Ieiri

**Affiliations:** 1 Department of Pediatric Surgery, Research Field in Medicine and Health Sciences, Medical and Dental Sciences Area, Research and Education Assembly, Kagoshima University, 8-35-1 Sakuragaoka, Kagoshima City, 890-8520 Japan; 2 Clinical Training Center, Kagoshima University Hospital, Kagoshima, Japan

**Keywords:** Communicating bronchopulmonary foregut malformations, Tracheoesophageal fistula, Esophageal atresia, Dextrocardia, Thoracoscopic repair, Suspending technique of anastomotic site

## Abstract

**Background:**

A communicating bronchopulmonary foregut malformation (CBPFM) group IB is very rare congenital malformation. Group IB is associated with tracheoesophageal fistula and esophageal atresia (TEF-EA) and a portion of one lung arisen from the esophagus (Gerle et al. in N Engl J Med. 278:1413–1419, 1968). The coexistence of TEF-EA and dextrocardia is also a rare and challenging setting for repair of TEF-EA. Therefore, the thoracoscopic surgery for TEF-EA require the technical devise because of the small operative space. We herein report a rare case of CBPFM group IB with intralobar sequestration of lung and a successful performing of thoracoscopic surgery for EA with dextrocardia in VACTERL association.

**Case presentation:**

A 2.2-kg term male neonate was born with an anal atresia, coarctation of the aorta, TEF-EA, renal anomalies, radial hemimelia, limb abnormalities (VACTERL association) and hypoplasia of the right lung with dextrocardia. The patient developed respiratory distress after admission. A two-stage operation for the TEF-EA was planned because of multiple anomalies and cardiac condition. In the neonatal period, esophageal banding at the gastroesophageal junction and gastrostomy were performed to establish enteral nutrition. After gaining body weight and achieving a stable cardiac condition, thoracoscopic surgery for TEF-EA was performed. The thoracoscopic findings revealed a small working space due to dextrocardia. To obtain a sufficient working space and to perform secure esophageal anastomosis, an additional 3-mm assistant port was inserted. To close the upper and lower esophagus, anchoring sutures of the esophagus were placed and were pulled to suspend the anastomotic site. Esophageal anastomosis was successfully performed. An esophagogram after TEF-EA surgery showed the connection between the lower esophagus and right lower lung. The definitive diagnosis was CBPFM group IB with intralobar sequestration. The thoracoscopic surgery was performed again for establishing oral intake. After transection of the bronchoesophageal fistula, the patient could perform oral feeding without pneumonia or respiratory distress.

**Conclusions:**

CBPFM type IB with intralobar sequestration is a rare condition. CBPFM type IB should be considered for a patients with respiratory symptom after radical operation for TEF-EA. In the present case, suspending the anastomotic site was effective and useful in thoracoscopic surgery for a TEF-EA patient with dextrocardia.

## Background

Communicating bronchopulmonary foregut malformation (CBPFM) group IB is very rare condition and difficult to obtain the early definitive diagnosis. CBPFM is defined as a congenital communication between the esophagus or stomach and an isolated portion of the respiratory tract. Group I is associated with tracheoesophageal fistula and esophageal atresia (TEF-EA), and a portion of one lung arisen from the esophagus is defined as group IB. The symptom and findings of CBPFM group IB are sometimes atypical. In addition, the coexistence of TEF-EA and dextrocardia is a rare condition and challenging setting for radical TEF-EA operation. The thoracoscopic surgery for TEF-EA with dextrocardia require determining an appropriate approach and technical devise because of the small operative space. We herein report successful thoracoscopic surgery for esophageal atresia of CBPFM group IB with dextrocardia.

## Case presentation

**Fig. 1:**
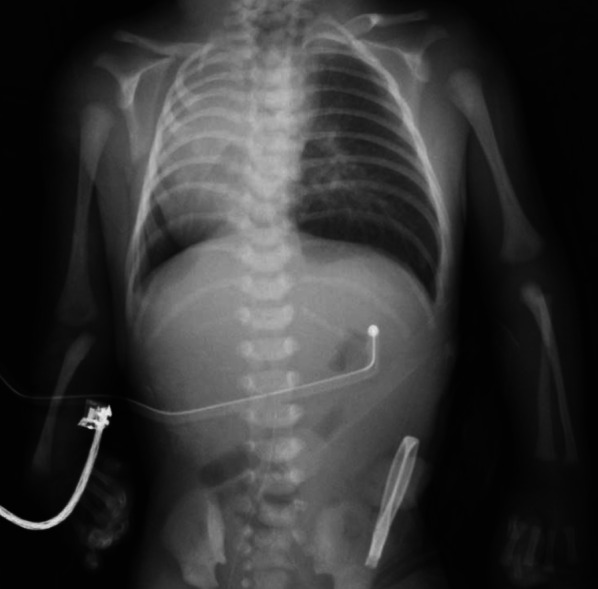
Chest X-ray findings after esophageal banding and gastrostomy was performed. The small right lung and mediastinal shift with dextrocardia and right radial hemimelia were detected

A 2.2-kg term male neonate was born with an anal atresia, coarctation of the aorta, TEF-EA, renal anomalies, radial hemimelia, limb abnormalities (VACTERL association) and the small right lung and mediastinal shift with dextrocardia. The patient developed respiratory distress and required a tracheal intubation and mechanical ventilation after admission. A two-stage operation for the TEF-EA was planned because of multiple associated anomalies including cardiac condition. In the neonatal period, esophageal banding at the gastroesophageal junction, gastrostomy and transverse colostomy were performed as palliative operations (Fig. 1). After gaining body weight and achieving a stable cardiac condition, thoracoscopic surgery for TEF-EA was performed. The patient was 2 months of age and his body weight and height were 3.3 kg and 47 cm. Aortic arch was confirmed by echocardiography before surgery and it was a normal left aortic arch. Coarctation of the aorta was not indicated for surgical management and it was managed conservatively.

**Fig. 2:**
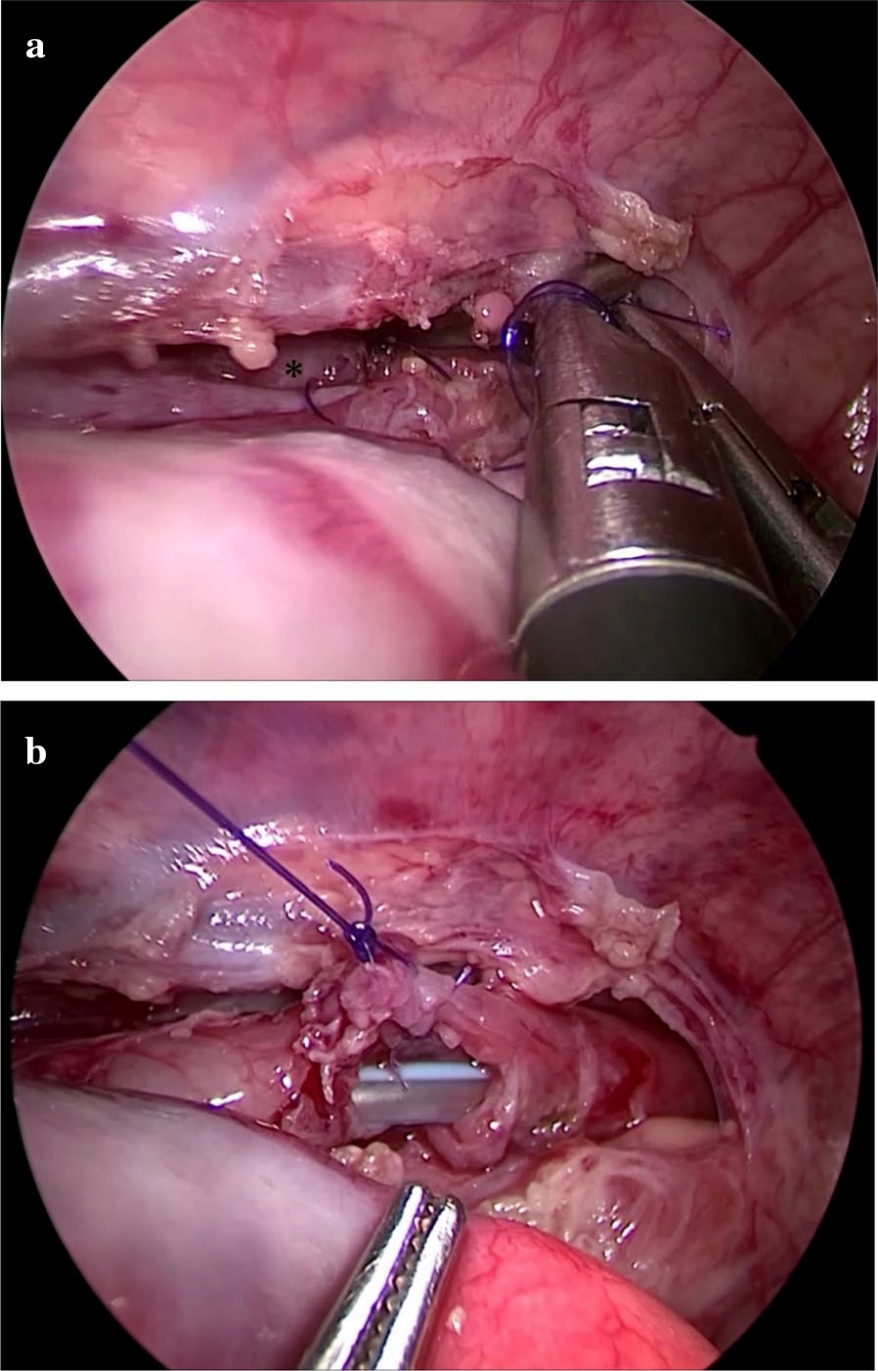
The thoracoscopic findings. **a** The thoracoscopic findings showed a small working space due to dextrocardia. Lower esophagus could be confirmed in the back of the operating field (asterisk). **b** After placed anchoring sutures of the esophagus. We could keep anastomotic site apart from the beating heart

Under general anesthesia with the patient in a supine position, broncho-fiberscopy was performed to confirm the location of the TEF initially. The TEF was recognized 8 mm proximal side from the bifurcation of the trachea and the bilateral opening of the peripheral bronchus were confirmed. Following bronchofiberscopy, the patient position was changed to a left three quarter prone position. The first port was inserted into the 5th intercostal space (ICS) on the posterior axillary line by an optical procedure. An artificial pneumothorax was established with 5 mmHg CO_2_ insufflation (1.5 L/min). And then, two additional ports were then inserted under thoracoscopic inspection: a 3-mm port at the 3rd ICS on the middle axillary line for operator’s right forceps and a 5-mm port at the 6th ICS on the middle axillary line for operator’s left forceps. The thoracoscopic findings showed a small working space because of dextrocardia. The right aortic arch was not recognized. The TEF was ligated using 5-0 absorbable trans-fixing sutures (PDSII; Ethicon, Cincinnati, OH, USA). To obtain a sufficient view and working space for performing precise esophageal anastomosis, an additional 3-mm assistant port was inserted at the 6th ICS on the posterior axillary line. A temporary approximating 5-0 PDS sutures were placed between the upper and lower esophagus (Fig. 2a). This suture was suspended from outside the thoracic wall using needle loop devise (Lapa-Her Closure, Hakko, Co. LTD, Tokyo, Japan) as shown in Fig. 2b. Under the combination of suspending suture of anastomotic site and the additional assistant forceps for compressing right lung, esophageal anastomosis were successfully performed using 6-0 PDS (PDSII; Ethicon, Cincinnati) interrupted sutures. And the laparotomy and removal of banding were performed. There were no intraoperative complications and the operating time was 207 min (Additional File 1).

**Fig. 3:**
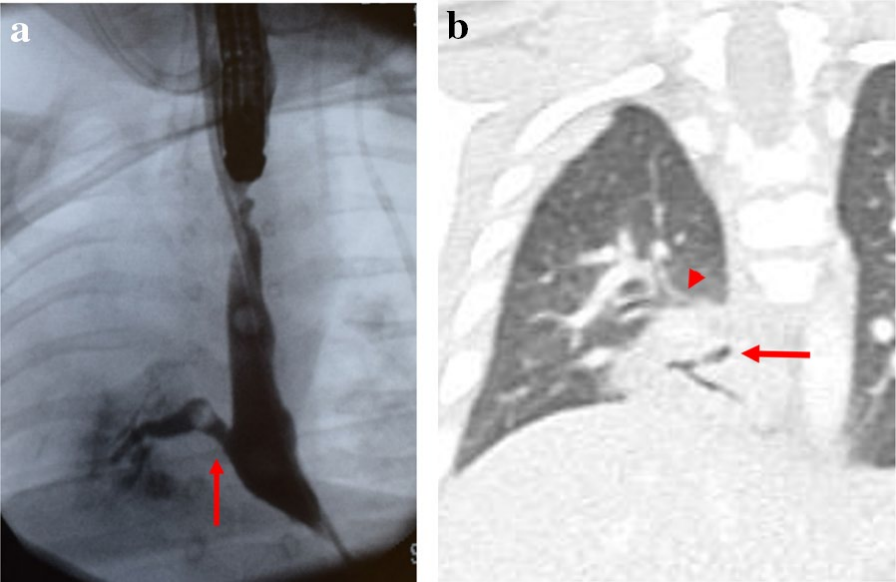
**a** An Esophagogram findings. The connection between the lower esophagus and right lower lung (arrow). **b** Computed tomography of the chest (coronal view). A connection between the esophagus and the right lower lung (arrow). Right main bronchus (arrowhead)

After operation, the patient required the ventilation due to a persistent atelectasis of the right lower lung and the atelectasis did not improve. A postoperative esophagogram 2 weeks after the operation showed minor leakage of anastomotic site but it was treated conservatively. A second esophagogram 2 months after the operation showed the connecting fistula between the lower esophagus and right lower lung (Fig. 3a). In a chest computed tomography (CT) examination, a non-aerated hypoplastic right lower lung and a connection between the esophagus and the right lower lung were recognized (Fig. 3b). However, distinct pulmonary vascular malformation or aberrant artery for the right lower lung could not be detected.

**Fig. 4:**
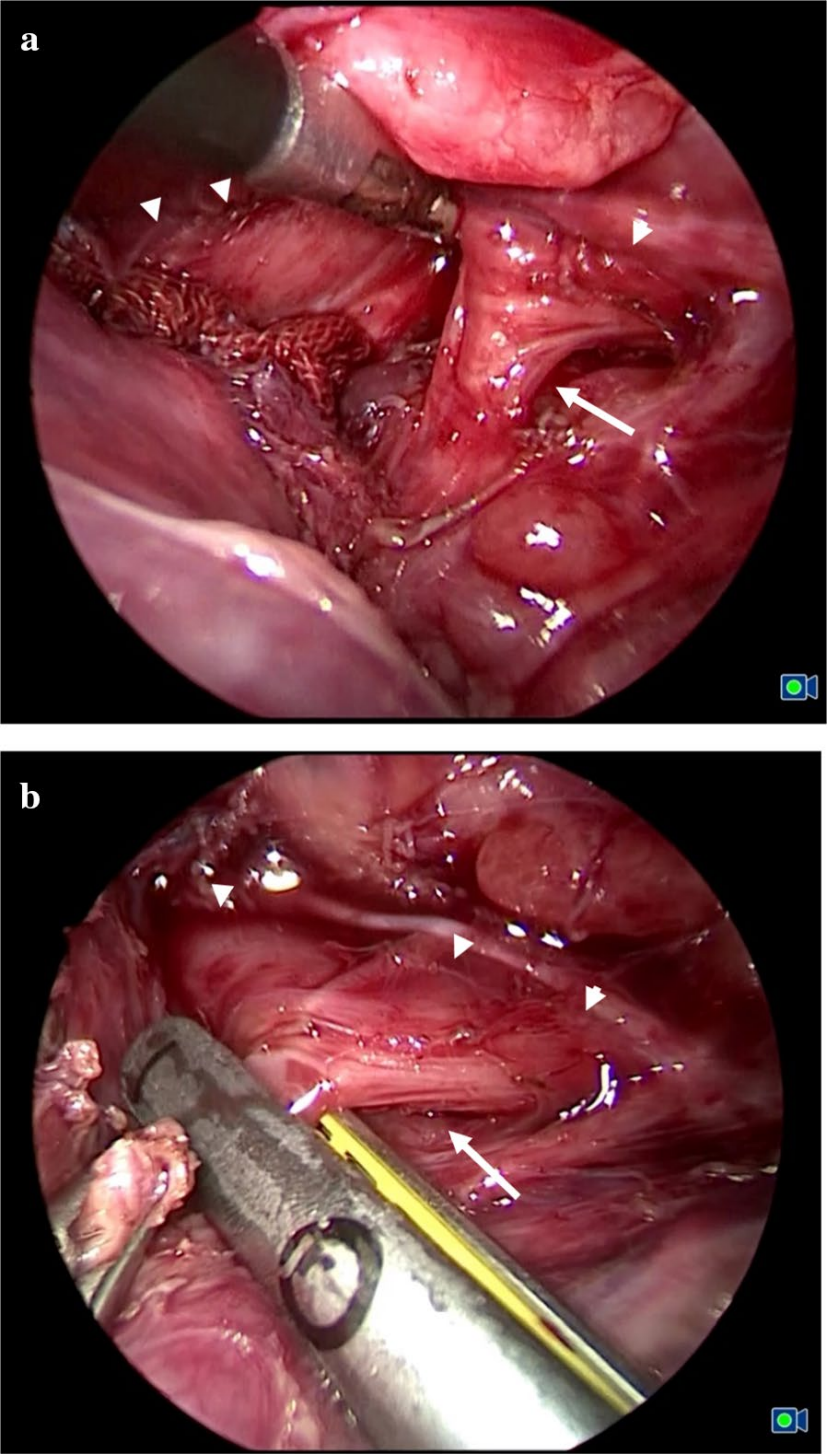
Thoracoscopic surgery for communicating bronchopulmonary foregut malformation. **a** Esophagus (arrowhead) and communication with the esophagus (arrow). **b** The bronchoesophageal fistula was ligated using a 5-mm stapler. Esophagus (arrowhead) and communication with the esophagus (arrow)

The definitive diagnosis was CBPFM group IB. In order to establish the early oral intake and to prevent regurgitation and pneumonia, thoracoscopic surgery for CBPFM group IB was performed. The thoracoscopic findings of the right lower lobe showed that the intralobar sequestration which was invested by visceral pleura. The bronchoesophageal fistula was expeditiously transected using a 5-mm stapler (JustRight Surgical/Bolder Surgical Holdings, Inc., Louisville, CO, USA) without removal of lung sequestration because of patient condition (Fig. 4a, b). The operating time was 194 min. A postoperative esophagogram of the entire esophagus did not show any leakage or residual fistula. The postoperative course was uneventful. After transection of the fistula, the patient could perform oral intake without pneumonia or respiratory distress. At 17 months old, the patient underwent laparoscopic assisted anorectoplasty for intermediate type anorectal malformation and then underwent closure of colostomy. Removal of lung sequestration would be planned and the patient is now preparing to undergo the operation.

## Discussion

CBPFM was first described by Gerle et al. in 1968 [1] and Srikanth et al. proposed an anatomical classification of CBPFM in 1992 [2]. The embryogenesis of CBPFM is unclear, however, CBPFM was suspected to occur from the defective development of a part of the lung buds and foregut because of a focal mesodermal defect. The aberrant lung tissue was separated with the residual tissue of the pulmonary and trachea during the rapid elongation of the esophagus, which accounted for the absence of part of the bronchial tree [3]. In our case, a combination of TEF-EA with the lower lobe connecting to the upper gastrointestinal tract were classified as CBPFM group IB. There were a few reports regarding CBPFM group IB, Srikanth et al. reviewed only 2 cases (3.9%) from 1959 to 1989 [2], and Yang et al. reviewed only 4 cases (6.5%) from 1992 to 2018 [4]. They also reported the mortality rate of group IB as 50% due to respiratory distress, postpneumonectomy syndrome and their associated anomalies. Early diagnose and careful treatment strategy should be required to improve their mortality rate.

The useful diagnostic modality for CBPFM is an esophagogram, however CBPFM group IB is difficult to diagnose because the presence of TEF-EA masked the lower pouch of esophagus. Eighty four percent cases with CBPFM group I were misdiagnosed and were initially operated for TEF-EA [4] and we were also initially misdiagnosed. In our case, respiratory distress still remained after the transection of TEF and chest x-ray imaging showed atelectasis of the right lung. A chest CT examination could detect an atelectasis of the right lower lung and connection of the right lower lung to lower esophagus. In the CBPFM group IB cases, the connection of the lung to the esophagus are partial, thus the respiratory symptoms of CBPFM group IB were less observed comparing with the CBPFM group IA. In our case, esophageal banding at the gastroesophageal junction was performed in the neonatal period, so his respiratory condition was stable and respiratory symptom did not became clinically evident because of the gastroesophageal reflux could not occur before radical operation for TEF-EA and removal of banding.

A surgical approach for CBPFM group IB should be modified according to a patient’s condition. In case with favorable condition of a patient, removal of affected lesion of lung should be recommended for radical operation. Nakaoka et al. reported that embolization for a bronchoesophageal fistula represents a palliative option for preventing regurgitation and pneumonia, when considering the poor condition of a patient or to salvage the affected lung [5]. In our case, the CBPFM lesion of the lung presented the intralobar sequestration type and his preoperative respiratory condition was not so stable that we could not perform resection of sequestration lung but only perform transection of the bronchoesophageal fistula. After transection of the bronchoesophageal fistula, the patient could perform oral feeding without pneumonia or respiratory distress. Two years had passed after the operation, he had not exhibited any respiratory symptom. We should observe carefully about his respiratory symptoms and wait for the removal operation for lung sequestration.

Thoracoscopic approaches to the treatment of TEF-EA have been introduced and recently became standard [6–8]. However the coexistence of EA and dextrocardia is a rare condition and challenging setting for radical TEF-EA operation. In addition, the left–right reversal of the organs increases the difficulty of operative procedures [9, 10]. In our case, the thoracoscopic findings showed a small working space compared with typical TEF cases because of the mediastinal shift with dextrocardia. The beating heart occupied the right thoracic cavity. To close the upper and lower esophagus, anchoring sutures of the esophagus were placed and were pulled to suspend the anastomotic site. The suspending suture at the anastomotic site was first reported by van der Zee et al. in 2012 [11]. In this report, stay suture was used for stabilizes the esophagus for suturing. We modified this technique for the TEF-EA with dextrocardia and we could keep anastomotic site apart from the beating heart. Using this suspending technique, sufficient view and space for anastomosis could be obtained.

## Conclusion

CBPFM group IB with intralobar sequestration is a rare condition. CBPFM group IB should be considered for a patients with respiratory symptom after radical operation for TEF-EA. In the present case, suspending the anastomotic site was effective and useful in thoracoscopic surgery for a TEF patient with dextrocardia.

## Supplementary Information

**Additional file 1.** The X-ray fingings showed radial hemimelia, limb abnormalities, the small right lung and mediastinal shift with dextrocardia.

## Data Availability

The datasets supporting the conclusions of this article are included within the article.

## Abbreviations

CBPFM: Communicating bronchopulmonary foregut malformation
TEF-EA: Tracheoesophageal fistula and esophageal atresia
ICS: Intercostal space
CT: Computed tomography

## References

[1] Gerle RD, Jaretzki A, Ashley CA, Berne AS (1968). Congenital bronchopulmonary-foregut malformation. Pulmonary sequestration communicating with the gastrointestinal tract. N Engl J Med..

[2] Srikanth MS, Ford EG, Stanley P, Mahour GH (1992). Communicating bronchopulmonary foregut malformations: classification and embryogenesis. J Pediatr Surg.

[3] Lee P, Westra S, Baba T, McCauley R (2006). Right pulmonary aplasia, aberrant left pulmonary artery, and bronchopulmonary sequestration with an esophageal bronchus. Pediatr Radiol.

[4] Yang G, Chen L, Xu C, Yuan M, Li Y (2019). Congenital bronchopulmonary foregut malformation: systematic review of the literature. BMC Pediatr.

[5] Nakaoka T, Uemura S, Yano T, Tanimoto T, Miyake H, Kasahara S (2009). Successful reconstruction of communicating bronchopulmonary foregut malformation associated with laryngotracheoesophageal cleft. J Pediatr Surg.

[6] Bax KM, van Der Zee DC (2002). Feasibility of thoracoscopic repair of esophageal atresia with distal fistula. J Pediatr Surg.

[7] Rothenberg SS (2002). Thoracoscopic repair of tracheoesophageal fistula in newborns. J Pediatr Surg.

[8] Wu Y, Kuang H, Lv T, Wu C (2017). Comparison of clinical outcomes between open and thoracoscopic repair for esophageal atresia with tracheoesophageal fistula: a systematic review and meta-analysis. Pediatr Surg Int.

[9] Smigiel R, Misiak B, Golebiowski W, Lebioda A, Dorobisz U, Zielinska M (2012). Esophageal atresia and anal atresia in a newborn with heterotaxia combined with other congenital defects. J Pediatr Genet.

[10] Rentea RM, Oyetunji TA, Erkmann J, Knowlton JQ, Hendrickson RJ (2017). Review of surgical and anesthetic management for esophageal atresia with tracheoesophageal fistula, unilateral pulmonary agenesis and dextrocardia. Pediatr Surg Int.

[11] van der Zee DC, Tytgat SH, Zwaveling S, van Herwaarden MY, Vieira-Travassos D (2012). Learning curve of thoracoscopic repair of esophageal atresia. World J Surg.

